# Supplementary material for: Eurasian jays do not copy the choices of conspecifics, but they do show evidence of stimulus enhancement
Source: PeerJ. 2016 Dec 1;4:e2746. doi: 10.7717/peerj.2746 (PMC5136130; doi:10.7717/peerj.2746)
Supplement: Data S1 — The number of object insertions (accidental and proficient) required per subject to solve the task (i.e., complete stage 3). The observer and control groups were trained following completion of Experiment 1. [file peerj-04-2746-s001.pdf]

Electronic Supplementary Material 1 for

Miller R, Logan CJ, Lister K, Clayton NS. 2016. Eurasian jays do not copy the choices of conspecifics, but they do show evidence of stimulus enhancement. *PeerJ* 4:e2746. doi:10.7717/peerj.2746.

Table S1. The number of object insertions (accidental and proficient) required per subject to solve the task (i.e., complete stage 3). The observer and control groups were trained following completion of Experiment 1.

**Group**	**ID**	**Sex**	**# of object insertions to solve the task**
Trained	Stuka	F	102
Trained	Dolci	F	136
Trained	Homer	M	111
Trained	Horatio	M	155
Trained	Jaylo	F	223
Trained	Poe	M	275
Observer	Cern	M	100
Observer	Booster	M	102
Observer	Gizmo	F	90
Observer	Lintie	F	139
Observer	Godot	M	119
Observer	Penny	F	135
Control	Chinook	F	104
Control	Dexter	M	137
Control	Roland	M	97
